# A Magnesium Transport Protein Related to Mammalian SLC41 and Bacterial MgtE Contributes to Circadian Timekeeping in a Unicellular Green Alga

**DOI:** 10.3390/genes10020158

**Published:** 2019-02-19

**Authors:** Helen K. Feord, Frederick E.G. Dear, Darren J. Obbard, Gerben van Ooijen

**Affiliations:** School of Biological Sciences, University of Edinburgh, Edinburgh EH9 3BF, UK; Helen.Feord@ed.ac.uk (H.K.F.); freddiedear@hotmail.co.uk (F.E.G.D.); Darren.Obbard@ed.ac.uk (D.J.O.)

**Keywords:** magnesium transport, circadian clocks, cellular rhythms, transporter proteins, *Ostreococcus tauri*

## Abstract

Circadian clocks in eukaryotes involve both transcriptional-translational feedback loops, post-translational regulation, and metabolic, non-transcriptional oscillations. We recently identified the involvement of circadian oscillations in the intracellular concentrations of magnesium ions ([Mg^2+^]_i_) that were conserved in three eukaryotic kingdoms. [Mg^2+^]_i_ in turn contributes to transcriptional clock properties of period and amplitude, and can function as a zeitgeber to define phase. However, the mechanism—or mechanisms—responsible for the generation of [Mg^2+^]_i_ oscillations, and whether these are functionally conserved across taxonomic groups, remain elusive. We employed the cellular clock model *Ostreococcus tauri* to provide a first study of an MgtE domain-containing protein in the green lineage. *Ot*MgtE shares homology with the mammalian SLC41A1 magnesium/sodium antiporter, which has previously been implicated in maintaining clock period. Using genetic overexpression, we found that *Ot*MgtE contributes to both timekeeping and daily changes in [Mg^2+^]_i_. However, pharmacological experiments and protein sequence analyses indicated that critical differences exist between *Ot*MgtE and either the ancestral MgtE channel or the mammalian SLC41 antiporters. We concluded that even though MgtE domain-containing proteins are only distantly related, these proteins retain a shared role in contributing to cellular timekeeping and the regulation of [Mg^2+^]_i_.

## 1. Introduction

Circadian clocks allow for the physiological anticipation of daily environmental changes (such as temperature and light) resulting from the Earth’s 24-h rotation on itself. These rhythms exist in most eukaryotes (both unicellular and multicellular organisms) and some prokaryotes [1]. The circadian clock controls daily rhythms through the working of a central oscillator. This oscillator modulates rhythmic outputs that control organismal and cellular physiology by taking into account environmental cues [2]. These rhythms are temperature compensated, persist in constant conditions, and have a period of approximately 24 h [3].

The study of circadian clocks has mainly focused on characterising transcriptional-translational feedback loops (TTFLs) [4]. These regulatory systems include positive and negative components which regulate each other and themselves, ultimately allowing for the regulation of many basic cellular processes over approximately 24 h. These loops are found across life; however, the genes involved in them differ between taxonomic groups [2]. This initially suggested that circadian clocks do not have a common origin. However, recent work has challenged this assumption, with experimental data suggesting the existence of non-transcriptional oscillators (NTOs) in eukaryotes [5,6]. An NTO was first discovered in prokaryotes; phosphorylation cycles of the cyanobacterial KaiC [7] protein persist in vitro in the absence of transcription or translation [8]. Evidence suggesting that NTOs exist in eukaryotes was provided by the observation that circadian rhythms in peroxiredoxin-oxidation states persist without transcription in human red blood cells [9] and the picoeukaryote *Ostreococcus tauri* [10]. Subsequently, peroxiredoxin oxidation cycles were used as a marker of universally conserved metabolic rhythms across Eukarya, Bacteria, and Archaea [11].

A further example of conserved cellular circadian rhythms came with the discovery that, in species representative of three distinct taxonomic lineages (animals, fungi, and plants), intracellular ion concentrations undergo daily oscillations [12,13]. Notably, daily oscillations of intracellular concentrations of Mg^2+^ ([Mg^2+^]_i_) were investigated in depth. [Mg^2+^]_i_ is low around dawn, increasing during the day to peak around dusk, and then decreasing during the night. These oscillations meet the classic hallmarks of circadian rhythms; they persist in constant conditions, have a cycle of approximately 24 h, are temperature-compensated, and entrain to relevant zeitgebers [3]. In this study, Mg^2+^ was also found to be a zeitgeber that regulates daily global translational rates through the highly Mg-sensitive mTOR [12]. The facts that [Mg^2+^]_i_ rhythms are circadian and control the transcriptional oscillator makes them a bona fide mechanistic clock component. Therefore, their in-depth study is warranted to further our understanding of conserved metabolic clocks.

Beyond the identification of the phenotype of daily [Mg^2+^]_i_ oscillations, the mechanisms responsible for them are only starting to be elucidated. The first step to study these daily ionic rhythms is to investigate the plasma membrane proteins that are responsible for temporally regulating Mg^2+^ transport in and out of the cell. To our knowledge, only two studies of Mg^2+^ transporting proteins exist within the context of circadian rhythms. Firstly, small interfering (siRNA)-mediated knockdown of the *SLC41A1* gene—a gene coding for a plasma membrane Na^+^/Mg^2+^ antiporter—was found to cause an increase in circadian period in human U2OS cells [12]. Secondly, PRL-2 (Phosphatase of Regenerating Liver 2), a known regulator of the CNNM proteins in mammalian cells, was found to regulate rhythmic [Mg^2+^]_i_ fluxes through diurnal expression [14]. Although Mg^2+^ oscillations exist in three eukaryotic kingdoms, the studies above are limited to mammals. Widening the study of plasma membrane transporter proteins to other species in the context of cellular rhythms could elucidate whether conserved proteins are at the basis of the conserved phenotypic oscillations across taxonomic groups.

The unicellular green alga *Ostreococcus* is a highly amenable circadian clock model with a simple cell structure [15], a reduced plant-like transcriptional clock architecture [16], and a gene-dense and non-redundant genome [17]. It has been a useful model to study eukaryotic cellular rhythms in general [10,11,12,18,19,20,21], as well as to study circadian clocks of the green lineage specifically [16,22,23]. To investigate if the roles of the Mg^2+^ transporting protein homologues are conserved between species in the regulation of the circadian clock, we here report a first study of a putative Mg^2+^-transporting protein related to the human SLC41A1 protein; *Ot*MgtE. Our results indeed show that this protein is involved in timekeeping and [Mg^2+^]_i_ regulation in the picoeukaryote *Ostreococcus*.

## 2. Materials and Methods

### 2.1. Ostreococcus tauri Cell Lines

Unless otherwise stated, *Ostreococcus* cultures were grown under 12 h/12 h light/dark cycles at 20 °C, under a blue light filter as previously described [10], and in artificial sea water supplemented with Keller media as described previously [12]. The clock marker line CCA1-LUC (Circadian Clock-Associated 1—Luciferase) was previously described [16], and an additional transgenic line was generated to overexpress the SLC41A1 homologue in *Ostreococcus* (ostta18g01947), here referred to as *Ot*MgtE. The gene was amplified using oligonucleotides catcctaggATGCGGGTCGCGTTCGAGC (forward) and catcctaggTCATACGAAGTGCTCAAAGA (reverse), and cloned by digestion with AvrII (NEB) and ligation into the pOtox vector [16]. Genomic transformation of the CCA1-LUC line was performed by our published method [24].

### 2.2. Verification of Overexpressing Lines

Bioluminescent imaging was performed to assess changes in circadian gene expression reported by CCA1-LUC, using a TriStar2 luminescent plate reader (Berthold Ltd., Bad Wildbad, Germany), fitted with red and blue LED lights with blue filter in white 384-well plates, as described earlier [12]. Each well contained 90 μL of algal cells with 1 mM D-luciferin, and imaging was performed under constant light (referred to as LL). Experiments testing the effect of cellular inhibitors were performed following the same method as previously published [10,12,19]. All period analyses were carried out using non-linear regression analysis in GraphPad Prism v.7.0 (GraphPad, San Diego, CA, USA) Data were normalised with a nonlinear regression fit and period was calculated between the hours 24 and 120 (or 12 and 160 in Figure 4) using a user-defined equation:

Y = (mX) + amplitude^(−kX)cos((2π(X-phase))/period)^, (1)

where X is time, Y is signal, and k is the decay constant (such that 1/k is the half-life), which is constrained to >0. This method has been described in more detail previously [25].

*OtMgtE* gene expression was assessed by quantitative polymerase chain reaction (qPCR). RNA was extracted using RNeasy Plant Mini Kits (Qiagen, Venlo, The Netherlands). Cells were pelleted, resuspended in artificial seawater, and lysed before following the manufacturer’s instructions. Samples were treated with DNase fromRNase-Free DNase Kit (Qiagen). Complementary DNA (cDNA) synthesis was performed using SuperScript II (Invitrogen, Waltham, MA, USA). qPCR was performed in a StepOnePlus machine with SYBERGREEN (Applied Biosystems, Waltham, MA, USA), using the *Ostreococcus EF1* (*Elongation factor 1*) gene (*ostta04g05410*) as a housekeeping gene. Oligonucleotides (Sigma-Aldrich, St. Louis, MO, USA) were: EF1-F: CCAGGCGGACGCCGGAATTT, EF1-R: CGCCGCTGATCCATGACGAC, OtMgtE-F: GGGCACGGACGATTTTAATCGGGC, and OtMgtE-R: TCGACGATGTGTTGAAAACG.

Quantification of intracellular Mg^2+^ was performed by ICP-MS (inductively coupled plasma mass spectrometry) and by a luminescent plate assay as previously described [12], with the exception of using an altered method with an assay buffer with 40 mM HEPES, 1 mM luciferin, 0.05 mg·mL QuantiLum (Promega, Madison, WI, USA), and 1 mM ATP [12].

### 2.3. Bioinformatics Analysis

Using the protein sequence of human SLC41A1 (Q8IVJ1), homologues were searched in other taxonomic groups using NCBI BLAST [26] (https://blast.ncbi.nlm.nih.gov/) (BLASTp, DELTA-BLAST, reseq_protein). For *Ulva mutabilis*, the BLAST was performed on the latest available proteome on the ORCAE website [27]. Proteins were then submitted to a Pfam [28] search to identify those with MgtE/CBS domains (Pfam10769/Pfam00571). NCBI BLAST searches were also repeated with a bacterial MgtE domain sequence. A maximum-likelihood tree (100 bootstraps) was generated to assess MgtE domain similarity using MEGA 7.0.25 [29]. The TMpred software (using default settings) was used to predict transmembrane domains of *Ot*MgtE [30]. 

## 3. Results

### 3.1. OtMgtE Overexpression Lengthens Circadian Period

**Figure 1:**
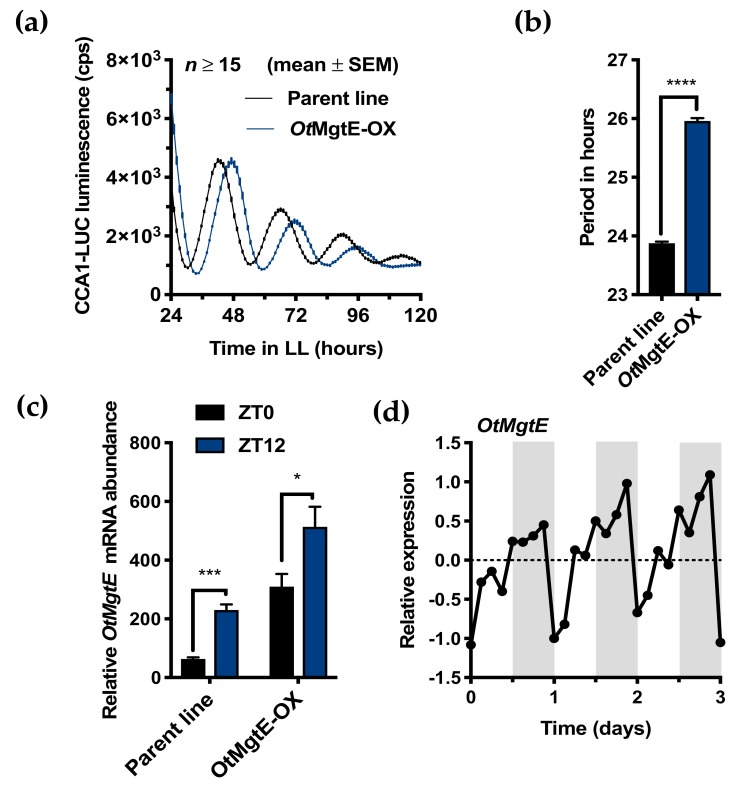
Overexpression of *OtMgtE* induces a long period phenotype. (**a**) CCA1 bioluminescent traces of *Ot*MgtE-OX (blue) compared to the parent line (black) in free running conditions (constant light). The graph shows a line through discrete time points, at a ~1-h sampling rate (mean ± Standard Error Mean (SEM)). (**b**) Free-running period inferred from traces in (a), student’s *t*-test, **** *p* < 0.0001. (**c**) Relative *OtMgtE* messenger RNA (mRNA) levels in the parent line compared to *Ot*MgtE-OX at ZT0 (dawn) and ZT12 (dusk). * = *p* < 0.05; *** = *p* < 0.001; student’s *t*-test. (**d**) Diurnal transcriptional expression profile of *OtMgtE* based on publicly available microarray data [31].

A list of *Ostreococcus* proteins identified as homologues of human Mg^2+^-transporting proteins in *Ostreococcus* was previously published [12], all of which are diurnally differentially regulated at the transcript level [31]. This list included a homologue for the human SLC41A1 protein, hereafter referred to as *Ot*MgtE, based on their shared MgtE domain (Supplementary Figure S1A). In addition, the prediction of five transmembrane domains based on hydrophobicity indicated that this protein is a transmembrane domain (Supplementary Figure S1B). *OtMgtE* was cloned into an overexpression vector and transfected into a parent line that expresses a translational fusion of CCA1 to luciferase [16]. Analysis of luminescent traces under constant light of the resultant algal lines compared to the parent line revealed that three transgenic lines exhibited an increase in circadian period (Supplementary Figure S2). Transgenic line 3 was selected for further study based on the effect size on period (~2 h) and amplitude of CCA1-LUC expression, and is referred to in this study as *Ot*MgtE-OX. *Ot*MgtE transcript levels in the overexpression line and parent line were increased at ZT12 (dusk) compared to ZT0 (dawn, Figure 1C), which is consistent with the known expression profile mined from publicly available microarray data (Figure 1D) [31]. In our line of expectations, a significant increase in *OtMgtE* expression was observed in the *Ot*MgtE-OX line at both time points. Together, these results indicate that *Ot*MgtE expression levels contribute to circadian period determination.

### 3.2. Effect of OtMgtE Overexpression on Intracellular Magnesium

**Figure 2:**
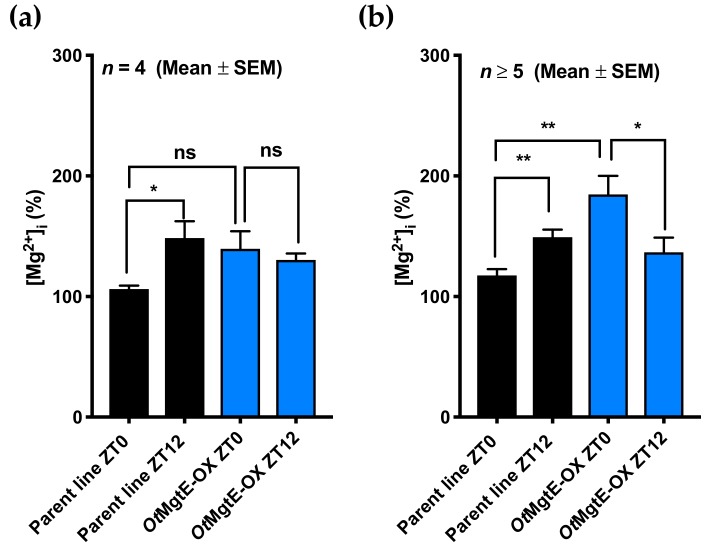
Overexpression of *OtMgtE* increases [Mg^2+^]_i_ at ZT0. Quantification of [Mg^2+^]_i_ by inductively coupled plasma mass spectrometry (ICP-MS) (**a**) or luciferase-based plate assays (**b**). A significant increase in [Mg^2+^]_i_ at dusk compared to dawn is observed in the parent line, and *OtMgtE* overexpression leads to an increase in [Mg^2+^]_i_ at ZT0. Non-significant (ns) = *p* > 0.05; * = *p* < 0.05; ** = *p* < 0.001; student’s *t*-test.

In the three eukaryotic cell types originally studied [12], [Mg^2+^]_i_ is low at dawn and high at dusk. We investigated how the overexpression of *OtMgtE* affects [Mg^2+^]_i_ at these time points. Using either ICP-MS (Figure 2A), or a luminescence-based plate assay (Figure 2B), we assessed [Mg^2+^]_i_ in cell extracts of the *Ot*MgtE-OX versus the parent line at dawn (ZT0) and dusk (ZT12). Although the relative effective size was variable between the different assays and replicate experiments, overexpression of *OtMgtE* consistently removed this trough levels of [Mg^2+^]_i_ observed in wild-type cells at ZT0 relative to ZT12. The differential diurnal regulation of intracellular magnesium observed in the wild-type or parent line was perturbed upon *OtMgtE* overexpression. We believe these results verify a role for the *Ostreococcus* homologue in transporting [Mg^2+^]_i_.

### 3.3. Treatment with Cobalt(III)hexamine and Low Extracellular Mg^2+^

**Figure 3:**
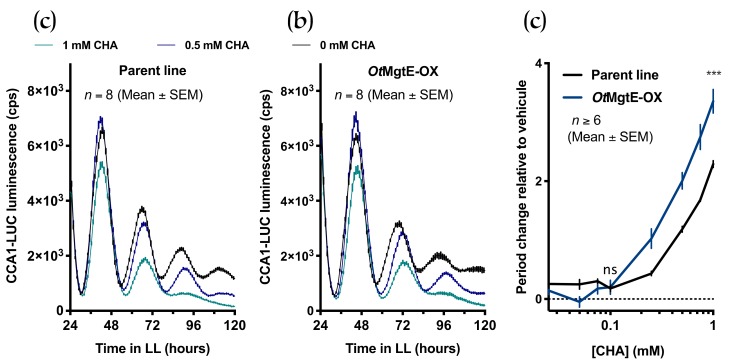
Overexpression of *OtMgtE* affects circadian period synergistically with cobalt(III)hexamine (CHA). Luminescent traces of the parent line (**a**) and *Ot*MgtE-OX (**b**) at certain CHA concentrations in constant light. The graph shows a line through discrete time points, at a ~1-h sampling rate (mean ± SEM). (**c**) The dose-response curve of circadian period for the parent and the *OtMgtE* overexpressing line at increasing concentrations of CHA. ns: *p* > 0.05 ***: *p* < 0.005; student’s *t*-test.

Cobalt(III)hexamine (CHA) blocks Mg^2+^ transport over biomembranes, and has previously been used to investigate the role of [Mg^2+^]_i_ in the cellular circadian clock. CHA treatment dose-dependently lengthened circadian period and increased [Mg^2+^]_i_ in both *Ostreococcus* and human cells [12]. We tested the combined effect of this inhibitor and *OtMgtE* overexpression to test whether these treatments exerted a combined effect (either additive or synergistic), or if they instead reversed their respective effects. Consistent with earlier observations, CHA dose-dependently lengthened circadian period in the parent line (Figure 3)**.** In the overexpression line, the same concentrations of CHA also induced a longer period. However, when the intrinsic period effect from overexpression was removed by plotting a full dose response relative to each line’s vehicle control, CHA clearly had a greater effect on period in the *Ot*MgtE-OX line than in the parent line (Figure 3C). These results show that CHA treatment and *OtMgtE* overexpression synergistically lengthen the circadian period. This result indicates that, like in human cells [32], CHA does not target SLC41A1 directly, and that overexpression and CHA treatment exert an effect on the circadian period independently, presumably by increasing [Mg^2+^]_i_. Based on this result, we can also infer that additional transmembrane proteins are likely to be involved in regulating the circadian period via [Mg^2+^]_i_.

**Figure 4:**
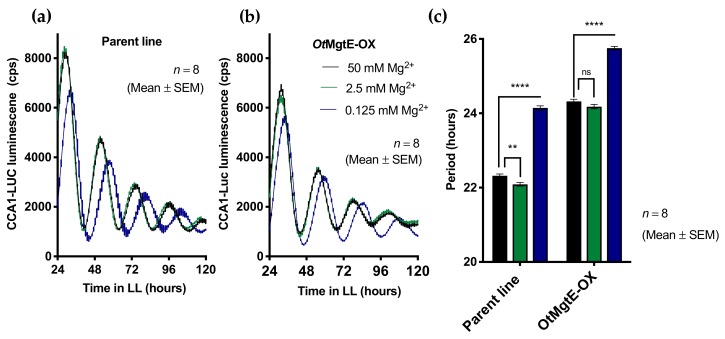
Overexpression of *OtMgtE* does not rescue the effect of low Mg^2+^ on the circadian period. Luminescent traces of (**a**) the parent line and (**b**) *Ot*MgtE-OX at different concentrations of extracellular Mg^2+^. The graph shows a line through discrete time points, at a ~1-h sampling rate (mean ± SEM). (**c**) Free-running period calculated from bioluminescent traces in (**a**,**b**). ns: *p* > 0.05 **: *p* < 0.01 ****: *p* < 0.0001; student’s *t* test.

We previously showed that decreased extracellular Mg^2+^ ([Mg^2+^]_e_) caused decreased [Mg^2+^]_i_ and dose-dependently lengthened the circadian period in *Ostreococcus* cells [12]. We tested the combined effect of the depletion of [Mg^2+^]_e_ and the overexpression of *OtMgtE*. *Ostreococcus* cells are normally grown in artificial seawater at a concentration of 50 mM [Mg^2+^]_e_; we tested how the circadian period of both lines was affected when incubated at 20 (2.5 mM [Mg^2+^]_e_) or 400 (0.125 [Mg^2+^]_e_) times less extracellular Mg^2+^ (Figure 4). As seen previously, an extreme reduction in [Mg^2+^]_e_ (0.125 mM) caused an increase in the circadian period in the parent line. Similarly, the effect on the *Ot*MgtE-OX line was also a ~2-h period increase. This result suggests that the effect of *Ot*MgtE on intracellular Mg^2+^ is not strong enough to counteract the extreme treatment of depleting [Mg^2+^]_e_ 400-fold. Note that the absolute values of free-running period differ between experiments in Figure 1; Figure 4, which is commonly observed in *Ostreoccoccus* and might result from batch-to-batch variations in the relative ionic composition of growth media, or from differences in the ages of cultures between experiments.

### 3.4. The MgtE Domains in OtMgtE and Animal SLC41 are Distinct

**Figure 5:**
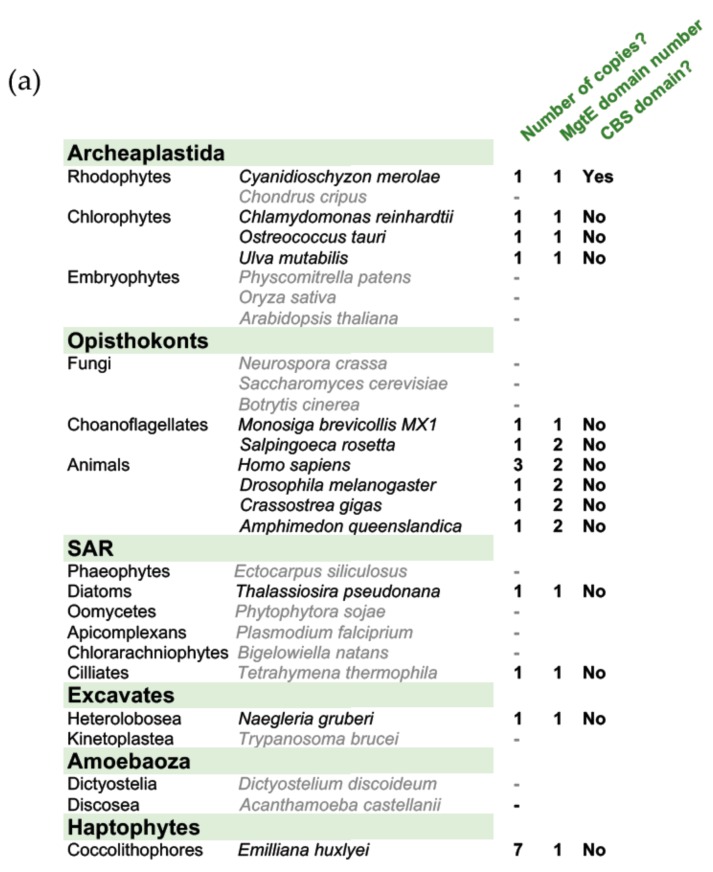

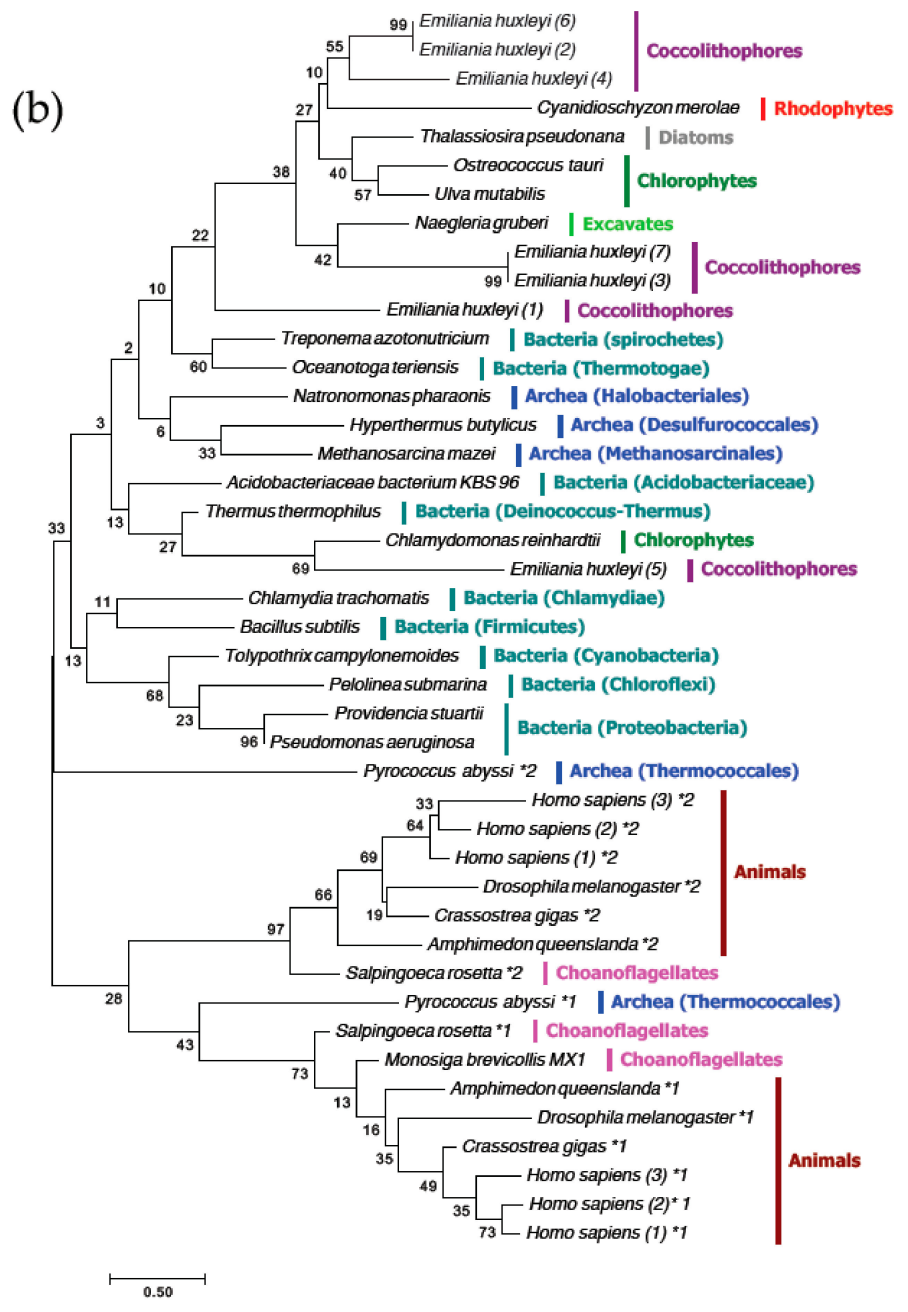
Phylogenetic comparison of *Ot*MgtE with eukaryotic and prokaryotic homologues. (**a**) Summary table of the distribution of proteins containing MgtE domains in eukaryotes and domain structure; we identified MgtE domains in species labelled in black but not those in grey. (**b**) Maximum-likelihood phylogenetic tree (based on 100 bootstraps) of MgtE domains in selected species. (x) denotes different homologue numbers from the same species, *x denotes the domain copy number within a homologue.

The mammalian SLC41 proteins were identified as Mg^2+^-transporting proteins based on a MgtE domain (Pfam10769) shared with bacterial MgtE proteins (Supplementary Figure S1A). Whereas bacterial MgtE proteins have one copy of this domain, eukaryotic SLC41 proteins have two copies, and archeal proteins have either one or two [33]. Using NCBI DELTA-BLAST homology searches on reference proteomes, we found no homologues of either MgtE/SLC41, nor of the bacterial MgtE domain alone in land plants, fungi, red seaweed (*Chondrus crispus*), or brown seaweed (*Ectocarpus siliculosus*; Figure 5A)**.** BLAST searches indicated that proteins with only a single MgtE domain exist in various eukaryotic taxonomic groups, including unicellular green algae (*Chlamydomonas reinhardtii, Ostreococcus tauri*), red algae (*Cyanidioschyzon merolae*), diatoms (*Thalassiosira pseudonana*), and multicellular green algae (*Ulva mutabilis*) (Figure 5A)**.** A maximum-likelihood phylogenetic tree was used to infer similarity between MgtE domains of relevant species (Figure 5B). MgtE domains from choanoflagellates, animals, and the archaeal species with duplicated domains are separated into two distinct groups. These two groups are distinct from bacteria, certain archaeal species, and non-opisthokont eukaryotic copies of the domain. The limited homology between the MgtE domains of *Ot*MgtE and mammalian SLC41 proteins indicates that, while they share a common ancestor, their conservation of function may be restricted.

**Figure 6:**
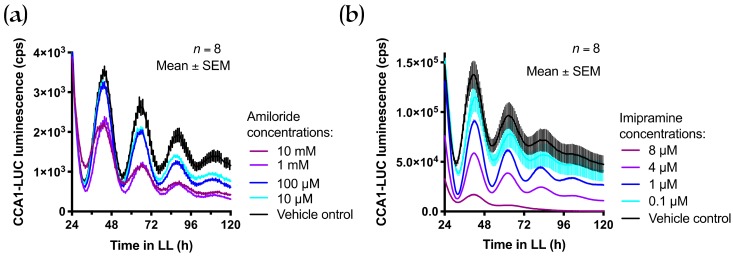
Effect of known inhibitors of SLC41 on CCA1 expression in *Ostreococcus tauri*. Luminescent traces of the CCA1 bioluminescent line (parent line) at a range of concentrations of (**a**) amiloride or (**b**) imipramine. The graph shows a line through discrete time points at a ~1-h sampling rate (mean ± SEM). LL: Constant light.

We performed pharmacological experiments to test the hypothesis that, unlike the mammalian SLC41 proteins, the *Ostreococcus tauri* homologue does not transport magnesium via Na^+^ antiporter activity. Amiloride is a commonly used compound to inhibit Na^+^-dependent Mg^2+^ transport [34,35]; we found no effect of this inhibitor on the circadian period in *Ostreococcus tauri* (Figure 6A). Imipramine is a cellular inhibitor that affects mammalian SLC41A1-mediated Mg^2+^ transport [32] and dose-dependently lengthens circadian rhythms in mammalian cells (John O’Neill, personal communication). Again, imipramine was not found to affect timekeeping in *Ostreococcus tauri* (Figure 6B). Given the remarkable conservation of effects that a plethora of cellular inhibitors exert on the circadian period between *Ostreococcus* and all other clock model organisms [10], the difference in response to SLC41 inhibitors supports the hypothesis that Na^+^-dependent transport might not play a role in timekeeping in *Ostreococcus*. Combined, phylogeny and pharmacology indicate that *Ot*MgtE may not be active as a Mg^2+^/Na^+^ antiporter like the mammalian SLC41 proteins.

## 4. Discussion

Discerning the plasma membrane Mg^2+^ transporter proteins responsible for daily intracellular Mg^2+^ oscillations is a first step in establishing the mechanisms that underlie daily intracellular magnesium rhythms across Eukarya. To this aim, it is important to investigate and compare proteins with putative homologues in various taxonomic groups to assess if the conserved phenotype observed is mediated by conserved cellular mechanisms. The human SLC41 family consists of three members [33]; SLC41A1 and SLC41A2 localise at the plasma membrane [32,36], while SLC41A3 mediates Mg^2+^ efflux at the mitochondrial membrane [37]. siRNA-mediated knockdown of SLC41A1 caused an increase in the circadian period. This effect was not additive with the effect of low extracellular Mg^2+^ [12], indicating that SLC41A1 exerts its contribution to the circadian period via Mg^2+^ transport. As [Mg^2+^]_i_ oscillations and the effects of CHA and quinidine on the circadian period are shared between human and algal cells, we extended our studies to an SLC41 homologue in *Ostreococcus tauri*.

Members of the mammalian SLC41 family of Na^+^/Mg^2+^ antiporters [33] were identified as Mg^2+^ transporters because they have two repeats of the MgtE domain. The *Ostreococcus* gene *Ostta18g01947* was identified as a candidate homologue based on the presence of a single MgtE domain. However, the duplicated animal MgtE domains are clearly distinct from those in other eukaryotic and bacterial proteins (Figure 5B). Bacterial MgtE and SLC41A1 have different modes of transporting Mg^2+^; the MgtE protein is a channel [38], while SLC41 is an antiporter exchanging Na^+^ for Mg^2+^ [39]. SLC41 proteins lack an N-terminal regulatory domain of MgtE, which could underlie differential modes of regulation [40]. The distinct clustering of MgtE domains (Figure 5B) suggests that the *Ostreococcus* protein may be more similar to the bacterial protein than to human SLC41 proteins. Supportive of that, amiloride and imipramine, which inhibit Na^+^/Mg^2+^ activity in mammalian cells [32,34,35], did not affect the circadian period in *Ostreococcus* cells (Figure 6). However, some results also argue against close conservation between bacterial MgtE and *Ot*MgtE. Firstly, bacterial MgtE proteins contain a CBS domain (named after the first protein it was identified in—Cystathionine ß Synthase [41,42]), which modulates Mg^2+^ transport in an ATP-dependent manner. With the single exception of the rhodophyte *Cyanidioschyzon merolae* protein, none of the eukaryotic proteins described in this study (including animal SLC41 proteins or *Ot*MgtE) contain a CBS domain. Secondly, CHA targets the bacterial MgtE protein [38], but not the human SLC41A1 proteins [32]. As CHA does not reverse the effect of *OtMgtE* overexpression, but rather acts synergistically (Figure 3C), CHA does not target *Ot*MgtE. CHA is also known to target the CorA Mg^2+^ transport protein in bacteria [43]. CorA is the ancestral protein of the eukaryotic MRS2 proteins; a protein family that exists in plants, animals, and fungi [44]. It is possible that (part of) the effect of CHA on the circadian period is mediated through its effect on MRS2 homologues in *Ostreococcus*, and further work investigating these homologues will be necessary to ascertain this.

All results together imply that *Ot*MgtE affects cellular timekeeping, similarly to the human SLC41A1 protein. As overexpression modulates cellular [Mg^2+^]_i_, a clear possibility is that the effects on timekeeping are mediated directly through differential [Mg^2+^]_i_. To our knowledge, extraneous to circadian clocks, not only is this the first study of a protein containing a MgtE domain outside prokaryotes or animals, it is also the first of any putative Mg^2+^ transporter in *Ostreococcus*. We provide evidence that additional, unidentified proteins are involved in regulating circadian rhythms via [Mg^2+^]_i_, providing an avenue for future studies to increase our understanding of the cellular mechanisms underlying daily Mg^2+^ fluxes.

## Supplementary Materials

The following are available online at https://www.mdpi.com/2073-4425/10/2/158/s1, Figure S1: (a) The *Ot*MgtE MgtE domain shares homology with the bacterial MgtE domain (here from *Thermus thermophiles*) and all three human SLC41 proteins as shown with this multiple sequence alignment. The two conserved motifs outlined by Wabakken et al. (2003) are shown in green. “*” denotes a fully conserved residue, “:” denotes conservation between groups of strongly similar properties and “.” denotes conservation between groups of weakly similar properties. (b) The hydrophobicity plot predicting transmembrane domains indicates that this protein is a transmembrane protein. Figure S2: Luminescent traces and period analysis of *Ot*MgtE overexpression lines. (a) Period analysis of 3 transgenic lines and parent line. (b) Luminescent traces: *Ot*MgtE overexpression induces a long period phenotype compared to the parent line in free running conditions (LL). Figure shows CCA1 bioluminescent traces over 4 days. Table S1: Protein accession numbers for sequences used in Figure 5.
