# Supplementary material for: A Magnesium Transport Protein Related to Mammalian SLC41 and Bacterial MgtE Contributes to Circadian Timekeeping in a Unicellular Green Alga
Source: Genes (Basel). 2019 Feb 19;10(2):158. doi: 10.3390/genes10020158 (PMC6410215; doi:10.3390/genes10020158)
Supplement: Supplementary file 1 [file genes-10-00158-s001.pdf]

(a)

```
OtMgtE         -TMLVGAGGNAGNQSAIHVIRGLATGEMENTNE-----CMRKTLVEQFQVGLM---LGVV     51
MgtE           VPVLLGTGGNTGNQSATLIIRALATRDLDLR-------dWRRVFLKEMGVGLL---LGLT     50
SLC41A3*1      VPPLVGLKGNLEMTLASRLSTAANTGQIDDPQE-----QHRVISSNLALIQVQATVVGLL     55
SLC41A2*1      VPALLGLKGNLEMTLASRLSTAVNIGKMDSPIE-----KWNLIIGNLALKQVQATVVGFL     55
SLC41A1*1      VPALLGLKGNLEMTLASRLSTAANIGHMDTPKE-----LWRMITGNMALIQVQATVVGFL     55
SLC41A3*2      TPVICGVGGNLVAIQTSRISTYLHMWSAPGVLPLQMKKFWPNPCSTFCTSEIN-------     53
SLC41A2*2      TPVINGIGGNLVAIQASRISTYLHLHSIPGELPD-EPKGCYYPFRTFFGPGVN-------     52
SLC41A1*2      TPVINGVGGNLVAVQASRISTFLHMNGMPGENSEQAPRRCPSPCTTFFSPDVN-------     53
                  : *  **     :  :                                :

OtMgtE         LSSAGFVRVLLTSPQGTDDLIG-------PFAIATALFAIVTT--------STCVGTALP     96
MgtE           LSFLLVGKVYWD---GHPLLLP-------VVGVSLVLIVFFAN--------LV--GAMLP     90
SLC41A3*1      AAVAALLL-GVV-SREEVDVAKVELLCA--SSVLTAFLAAFALG---------VLMVCIV     102
SLC41A2*1      AAVAAIIL-GWI-PEGKYYLDHSILLCS--SSVATAFIASLLQG---------IIMVGVI     102
SLC41A1*1      ASIAAVVF-GWI-PDGHFSIPHAFLLCA--SSVATAFIASLVLG---------MIMIGVI     102
SLC41A3*2      SMSARVLLLLVV-PGHLIFFYIIYV-EGQSVINSQTFVVLYLLAGLIQVTILLYLAEVMV     111
SLC41A2*2      NKSAQVLLLLVI-PGHLIFLYTIHLMKSGHTSLTIIFIVVYLFGAVLQVFTLLWIADWMV     111
SLC41A1*2      SRSARVLFLLVV-PGHLVFLYTISCMQGGHTTLTLIFIIFYMTAALLQVLILLYIADWMV     112
                    .             .                :.                    :

OtMgtE         FLLMRLNQDPANAGTSVQVVM-DVSGVIIT------   125
MgtE           FLLRRLGVDPALVSNPLVATLSDVTGLLIYL-----   121
SLC41A3*1      IGARKLGVNPDNIATPIAASLGDLITLSILA-----   133
SLC41A2*1      VGSKKTGINPDNVATPIAASFGDLITLAILA-----   133
SLC41A1*1      IGSRKIGINPDNVATPIAASLGDLITLALL------   132
SLC41A3*2      RLTWHQALDPDNHCIPYLTGLGDLLGSS--------   139
SLC41A2*2      HHFWRKGKDPDSFSIPYLTALGDLLGTALLA-----   142
SLC41A1*2      HWMWGRGLDPDNFSIPYLTALGDLLGTGLLAXSLCA   148
                       :*        . : *:
```

(b)

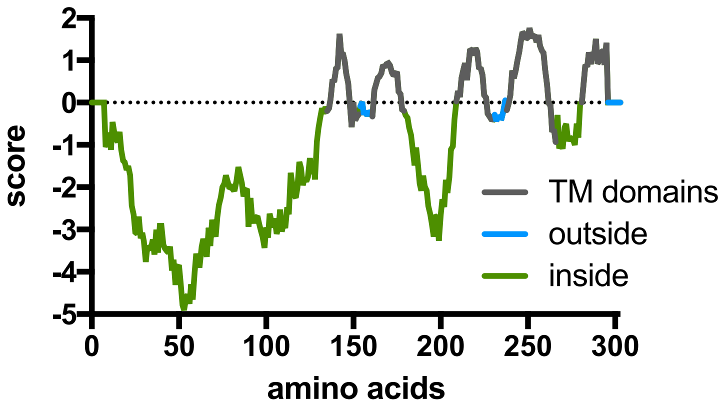

Supplementary Figure 1: **(a)** The *Ot*MgtE MgtE domain shares homology with the bacterial MgtE domain (here from *Thermus thermophiles*) and all three human SLC41 proteins as shown with this multiple sequence alignment. The two conserved motifs outlined by Wabakken *et al.* (2003) are shown in green. * denotes a fully conserved residue, : denotes conservation between groups of strongly similar properties and . denotes conservation between groups of weakly similar properties. **(b)** The hydrophobicity plot predicting transmembrane domains indicates that this protein is a transmembrane protein.

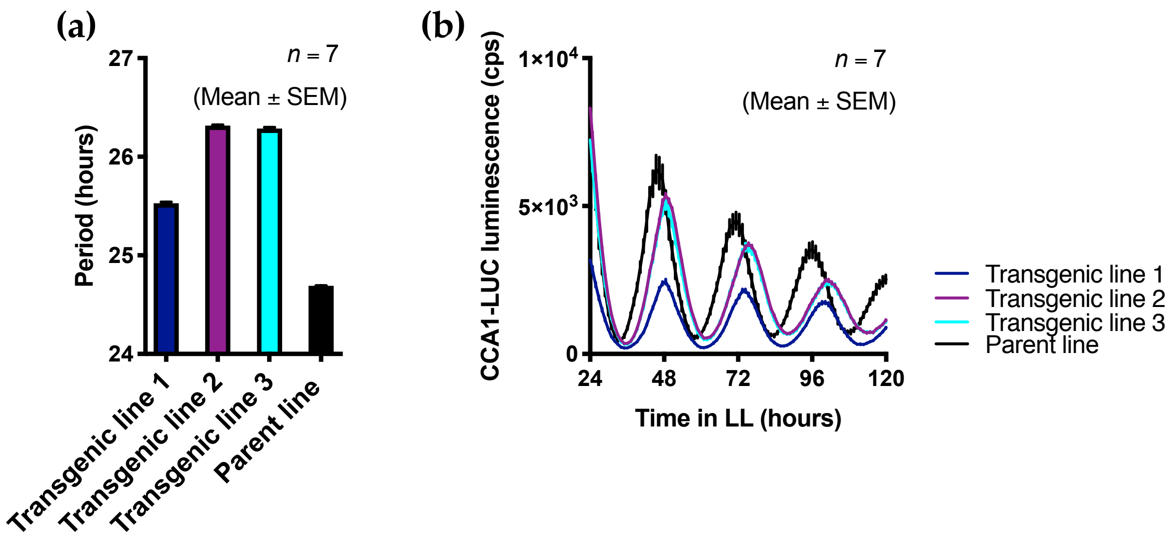

Supplementary Figure 2: Luminescent traces and period analysis of OtMgtE overexpression lines. (a) Period analysis of 3 transgenic lines and parent line. (b) Luminescent traces: OtMgtE overexpression induces a long period phenotype compared to the parent line in free running conditions (constant light). Figure shows CCA1 bioluminescent traces over 4 days.

Supplementary Table 1: Protein accession numbers for proteins used in figure 4

Species (NCBI taxonomic ID)	NCBI/Uniprot accession number, (homolog number where >1)
Homo sapiens (9606)	Q8IVJ1 (1)<br>(SLC41A2) Q96JW4<br>(SLC41A3) Q96GZ6
Drosophila melanogaster (7227)	M9PHE6
Crassostrea gigas (29159)	K1QPN0
Amphimedon queenslandica (400682)	XP_019864491.1
Monosiga brevicollis MX1 (431895)	A9VBR3
Salpingoeca rosetta (946362)	F2UAH4
Chlamydomonas reinhardtii (3055)	A8J2E0
Ostreococcus tauri (70448)	A0A096P9B9
Emiliania huxleyi (2903)	(1) R1EK90<br>(2) R1DRZ5<br>(3) R1FDF9<br>(4) R1CTW0<br>(5) R1CBM8<br>(6) R1EBL0<br>(7) R1F8I7
Thalassiosira pseudonana (35128)	B8CF51
Cyanidioschyzon merolae (45157)	M1V9W9
Naegleria gruberi (5762)	D2VNX4
Chlamydia trachomatis (*813)*	A0A0E9C5A4
Pseudomonas aeruginosa (208964)	Q9I544
Bacillus subtilis (224308)	O34442
Providencia stuartii (471874)	B2PYL8
Tolypothrix campylonemoides (1136105)	WP_071838811.1
Treponema azotonutricium (545695)	F5YDZ2
Pelolinea submarina (913107)	WP_116224794.1
Thermus thermophiles (274)	Q5SMG8
Acidobacteriaceae bacterium KBS 96 (1267535)	WP_020720448.1
Oceanotoga teriensis (515440)	WP_109604606.1
Natronomonas pharaonic (348780)	A0A1U7EW96
Pyrococcus abyssi (272844)	Q9UZ17
Hyperthermus butylicus (54248)	WP_048061491
Methanosarcina mazei (2209)	A0A0F8MPI8

Ulva mutabilis proteome accessed from ORCAE, gene *ID:* UM021_0210.1
